# Salidroside Regulates Energy Metabolism Reprogramming to Suppress Tumor Proliferation: Mechanisms and Therapeutic Implications

**DOI:** 10.3390/metabo16100748

**Published:** 2026-10-05

**Authors:** Haolin Wang, Yuxin Du

**Affiliations:** 1 Department of Chinese Medicine, Beijing University of Chinese Medicine, Liangxiang Campus, Fangshan District, Beijing 102488, China; whlphd@126.com; 2 School of Medicine, Xizang Minzu University, No.6, Wenhui East Road, Weicheng District, Xianyang 712082, China

**Keywords:** salidroside, cancer metabolism reprogramming, glycolysis, lipid metabolism, signaling transduction pathway

## Abstract

Salidroside, a phenylethanol glycoside and principal bioactive constituent of *Rhodiola rosea*, has emerged as a promising multi-target anti-tumor agent with significant effects on cancer energy metabolism. This review summarizes and analyzes the mechanisms it uses to reprogram tumor energy metabolism. Recent studies show that it upregulates the tumor suppressor miR-1343-3p in gastric cancer, which in turn targets two distinct metabolic axes: the OGDHL/PDHB–pyruvate axis, which impairs pyruvate oxidative decarboxylation and tricarboxylic acid cycle flux, and the ACOT11/FFA axis, which disrupts fatty acid hydrolysis and lipid-derived energy production. Both pathways are likely to converge at the reduction in acetyl-CoA, decreasing ATP synthesis and lowering the energy supply essential for rapid tumor proliferation. Furthermore, salidroside inhibits glycolysis through regulating STAT3/c-Myc, SLC7A11/cystine/GSH/GPX4, and PI3K/Akt/mTOR signaling pathways and sensitizes cancer cells to ferroptosis via regulating lipid metabolism and iron homeostasis. In vivo and in vitro studies indicate that salidroside possesses broad anti-tumor activity against a number of malignancies. This review synthesizes the findings from the past decade, providing a framework for understanding the multi-targeted inhibitory effects of salidroside on tumor metabolism and the challenges for clinical translation.

## 1. Introduction

### 1.1. Cancer Metabolism

Cancer cells exhibit explicit alterations in cellular metabolism that distinguish them from normal cells. The concept of metabolic reprogramming of malignancy was established by Warburg’s seminal observation that tumor cells preferentially utilize glycolysis for ATP production even under aerobic conditions—a phenomenon now universally recognized as the Warburg effect [1]. In addition to glucose metabolism, recent research has revealed that cancer cells rewire glutamine, fatty acid, and nucleotide metabolism to meet the biosynthetic and bioenergetic demands for rapid cancer cell proliferation, invasion and metastasis [2,3].

Tumor metabolic reprogramming is not merely a passive consequence of oncogenic transformation but also an active driver of malignant progression. The tricarboxylic acid (TCA) cycle, traditionally viewed as an oxidative pathway for ATP generation, becomes “truncated” in many tumors, through intermediates diverted to macromolecular synthesis [4]. Recent studies have verified the coordinated interaction between glycolysis and the TCA cycle, wherein pyruvate and lactate can be recycled to sustain mitochondrial function while supporting biosynthesis [5]. This metabolic plasticity provides both challenges and opportunities for therapeutic intervention.

MicroRNAs (miRNAs) have emerged as critical regulators of tumor metabolism through post-transcriptional regulation of metabolic enzyme expression [6]. By binding to complementary sequences in target mRNAs, miRNAs can cause coordinated changes in metabolites across the whole metabolic pathway. The tumor suppressor miR-1343-3p, initially characterized for its role in inhibiting oncogenic transcription factors, has recently been identified as a key regulator of cancer energy metabolism [7,8,9].

### 1.2. Salidroside: A Bioactive Compound with Multiple Effects

Salidroside is a phenylethanol glycoside isolated from the roots of *Rhodiola rosea* (Crassulaceae), a plant traditionally used in Tibetan medicine for its adaptogenic properties [10]. The compound is characterized by a hydroxybenzene ring linked to a β-D-glucoside moiety, with the phenolic structure considered as a key pharmacophore for its biological activity [11,12].

Over the past two decades, salidroside has clearly demonstrated a diverse spectrum of pharmacological effects, including antioxidant, anti-inflammatory, anti-fatigue, neuroprotective, cardioprotective, and immunomodulatory activities [13,14]. Recently, substantial evidence has proved its anti-tumor properties. In vivo and in vitro studies have shown that salidroside exerts multifaceted anti-tumor effects, leading to the inhibition of proliferation, migration, and invasion of some cancers [15,16,17,18,19,20,21,22]. Although the anti-tumor effects of salidroside have been proved, the mechanisms underlying its impact on tumor energy metabolism have not been elucidated. This review aims to summarize current studies on the regulation of tumor energy metabolism by salidroside. All eligible studies that investigated the effects of salidroside on tumor metabolism are included. To identify cancers and interventions, both Medical Subject Headings (MeSH) and free-text keywords were employed in the search strategy as follows: Salidroside OR Rhodioloside OR Rhodiola rosea OR Rhodiola crenulata; Neoplasm OR Tumor OR Cancer and Metabolism. A systematic literature search across PubMed, Web of Science, and ScienceDirect was conducted, covering all the original literature published before August 2026. The following inclusion criteria were systematically applied: in vitro and in vivo experiments; studies investigating the effects of salidroside on cancer metabolism; and studies presenting original, independent data with full-text accessibility. The following exclusion criteria were systematically applied: investigations involving additional pharmacological agents and the absence of a comparator group. By analyzing current findings, this review aims to provide a mechanistic and translational framework to promote our understanding of the effects of salidroside on tumor metabolism reprogramming.

## 2. Salidroside Mediating Tumor Glucose Metabolism Reprogramming

### 2.1. The miR-1343-3p/OGDHL/PDHB Axis

A key finding for understanding the metabolic effects of salidroside was the regulation of glucose metabolism through the miR-1343-3p/OGDHL/PDHB axis in two gastric cancer cells—MGC 803 and AGS [8]. High-throughput sequencing analysis of gastric cancer cells treated with salidroside identified 44 differentially expressed miRNAs, among which miR-1343-3p exhibited the most significant upregulation [7,8,9]. Bioinformatic analysis revealed that miR-1343-3p negatively correlates with α-ketoglutarate dehydrogenase-like (OGDHL), a key component of the α-ketoglutarate dehydrogenase complex in the TCA cycle. RNA immunoprecipitation and rescue experiments further confirmed that miR-1343-3p directly targets OGDHL in gastric cancers [8].

OGDHL catalyzes the oxidative decarboxylation of α-ketoglutarate to succinyl-CoA, maintaining TCA cycle homeostasis and cellular energy production [23]. In gastric cancer, OGDHL was found to interact with pyruvate dehydrogenase E1 subunit β (PDHB), a core component of the pyruvate dehydrogenase complex that converts pyruvate to acetyl-CoA [24]. This interaction between OGDHL and PDHB was clarified by co-immunoprecipitation assays, revealing an enzyme complex that links two key metabolic subunits in gastric cancer cells [8].

Further experiments indicated that salidroside treatment upregulates miR-1343-3p, which directly targets OGDHL mRNA, reducing OGDHL protein levels in gastric cancer cells. With the reduction in OGDHL, the degradation of PDHB protein increases. Since the causal link between OGDHL reduction and PDHB destabilization has not yet been fully proven, the study requires further clarification. Although PDHB mRNA levels remain unchanged, pyruvate oxidative decarboxylation decreased. Accordingly, acetyl-CoA production decreases, TCA cycle flux is impaired, and ATP synthesis declines [8]. This pathway showed that salidroside probably intervenes in glycolysis and mitochondrial oxidative phosphorylation in gastric cancer.

The clinical relevance of this mechanism is supported by analysis of the TCGA database, which shows that both OGDHL and PDHB are significantly upregulated in gastric cancer tissues compared to normal gastric mucosa [8]. Kaplan–Meier survival analysis further demonstrated that gastric cancer patients with high expression of OGDHL or PDHB have significantly shortened survival periods, implicating these enzymes as possible prognostic markers [8].

A functional rescue experiment using miR-1343-3p mimics and inhibitors suggested the significance of this regulatory axis in gastric cancer cells. These mimics enhanced the effects of salidroside treatment—reducing OGDHL expression, decreasing PDHB protein levels, and attenuating ATP production—while miR-1343-3p inhibitors reversed these effects [8]. Furthermore, OGDHL-targeted siRNA treatment synergized with salidroside in inhibiting cell proliferation, indicating OGDHL as a key glycolysis effector in gastric cancer cells [8].

### 2.2. Inhibition of Glycolysis Through JAK2/STAT3/c-Myc Signaling

The JAK2/STAT3/c-Myc pathway is a vital regulator of tumor metabolism, promoting glycolysis through transcriptional upregulation of glycolytic enzymes and glucose transporters [25,26,27]. c-Myc is a well-established key regulator of glycolysis, transcriptionally activating glucose transporters (GLUT1), hexokinase 2 (HK2), lactate dehydrogenase A (LDHA), and other glycolytic enzymes [28,29]. Some natural products have been reported to inhibit the STAT3 signaling pathway and have exhibited anti-tumor activities in vitro and in vivo [30,31]. Salidroside has proved to inhibit STAT3 phosphorylation in some cancers [32,33,34].

In ovarian cancer, salidroside inhibits glycolysis through regulating the STAT3/c-Myc signaling pathway, providing a complementary mechanism to the anti-tumor effect of salidroside [34]. Salidroside treatment reduced glucose consumption, lactate production, and ATP generation in a dose-dependent manner, indicating the potent inhibition of glycolytic flux in ovarian cancer cells [34]. Western blot further demonstrated that salidroside reduced phosphorylated STAT3 and c-Myc expression in ovarian cancer cells. The addition of a STAT3 agonist, Colivelin TFA, weakened salidroside’s effects, suggesting that salidroside functions through STAT3/c-Myc pathway inhibition [34]. By suppressing c-Myc expression, salidroside reduces the expression of key glycolytic regulators including GLUT1, HK2, and LDHA in ovarian cancer cells [34].

### 2.3. PI3K/Akt/mTOR Pathway Regulation

The PI3K/Akt/mTOR signaling pathway is a central regulator of cellular metabolism, controlling glucose uptake, glycolysis, and lipid synthesis [35]. mTOR activation promotes glycolysis through hypoxia-inducible factor (HIF-1α) and stimulates lipid synthesis through sterol regulatory element-binding protein (SREBP) activation [36]. In colon and breast cancer, salidroside suppressed the PI3K/Akt/mTOR signaling pathway, inhibiting the proliferation of cancer cells [37,38]. By decreasing the PI3K/Akt/mTOR pathway, salidroside is supposed to reduce the glycolysis in cancers, indicating a possible mechanism of metabolic regulation by salidroside.

## 3. Salidroside Intervening in Tumor Lipid Metabolism Reprogramming

### 3.1. The miR-1343-3p/ACOT11/FFA Axis

Lipid metabolism reprogramming plays an important role in cancer growth, providing energy, membrane components, and signaling molecules essential for proliferation [39]. Recent study from the same group has suggested that salidroside disrupts tumor lipid metabolism through the miR-1343-3p/ACOT11/FFA axis in gastric cancer cells, providing a mechanism complementary to its effects on glucose metabolism [8,9].

Acyl-CoA thioesterase 11 (ACOT11) is a lipid metabolism enzyme that hydrolyzes long-chain fatty acyl-CoA to free fatty acids (FFAs) and Coenzyme A, and then FFA undergoes mitochondrial β-oxidation to generate acetyl-CoA [40,41]. This reaction is significant for regulating fatty acid utilization and energy release from lipid stores.

In gastric cancer, ACOT11 expression was found to be negatively correlated with miR-1343-3p expression, suggesting miR-1343-3p-mediated lipid metabolism regulation. RNA immunoprecipitation assays verified the interaction between miR-1343-3p and ACOT11 mRNA in gastric cancer cells, indicating ACOT11 as a target for miR-1343-3p [9].

Further experiments indicated that salidroside upregulates miR-1343-3p, which binds ACOT11 mRNA, leading to reduced ACOT11 expression at both mRNA and protein levels [9]. ACOT11 downregulation causes the accumulation of the substrate, fatty acyl-CoA, which accordingly decreases subsequent metabolic products: free fatty acids and acetyl-CoA. These metabolic changes result in reduced ATP production and impaired cell proliferation in gastric cancer [9].

In vivo gastric cancer xenograft models suggested that salidroside significantly inhibited tumor growth while reducing ACOT11 expression, free fatty acids, and acetyl-CoA and ATP levels in tumor tissues [9]. These findings establish the miR-1343-3p/ACOT11/FFA pathway as a possible mechanism by which salidroside disrupts tumor energy metabolism.

### 3.2. Convergence of Glucose and Lipid Metabolism Pathways

**Figure 1:**
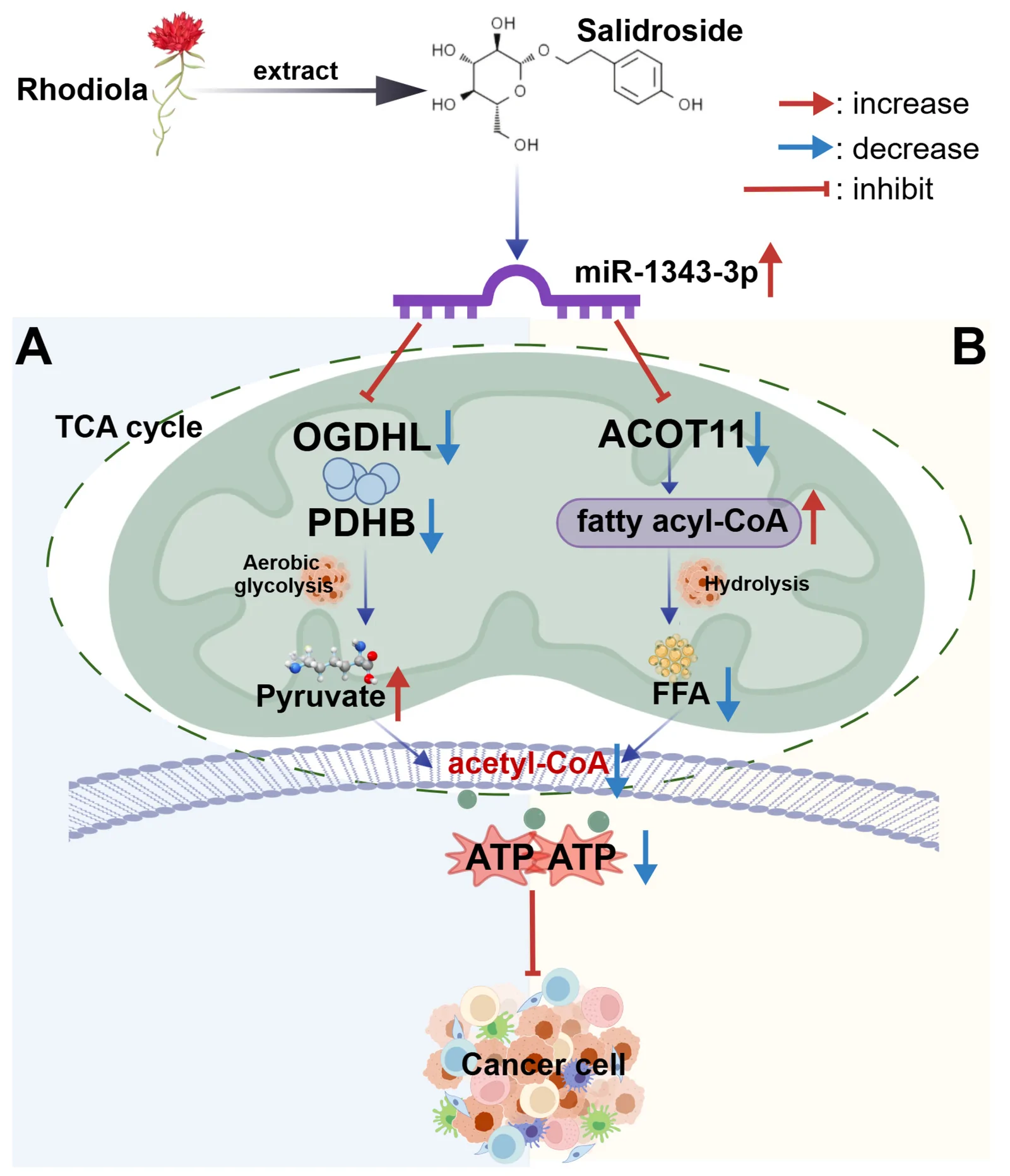
Mechanism of salidroside’s regulation of glucose and lipid metabolism through miR-1343-3p in gastric cancers. Salidroside upregulates miR-1343-3p—which targets and downregulates OGDHL/PDHB—interfering with the conversion of pyruvate to acetyl-CoA (**A**), and targets and downregulates ACOT11—which causes the accumulation of fatty acyl-CoA, and reduces free fatty acids and acetyl-CoA accordingly (**B**). Both pathways converge on acetyl-CoA reduction, which in turn reduces to the TCA cycle, with ATP synthesis eventually declining.

Based on the documented experiments, a speculative mechanism of salidroside is the convergence of its effects on glucose and lipid metabolism at the point of acetyl-CoA production in gastric cancer [8,9]. As shown in Figure 1, salidroside-induced upregulation of miR-1343-3p simultaneously targets OGDHL/PDHB—a glucose metabolism pathway—interfering with the conversion of pyruvate to acetyl-CoA, and ACOT11—a lipid metabolism pathway—causing fatty acyl-CoA accumulation, and reduces free fatty acids and acetyl-CoA products accordingly in gastric cancer [8,9].

The less acetyl-CoA that is produced, the less enters the TCA cycle, and the less ATP that is generated. Acetyl-CoA is likely to serve as the central metabolic point connecting carbohydrate and lipid catabolism to mitochondrial energy production in gastric cancer. By targeting the input at this point, salidroside effectively “starves” cancer cells with the decreased substrate necessary for oxidative phosphorylation, by way of reducing the production of acetyl-CoA for subsequent biosynthetic processes [8,9].

### 3.3. Ferroptosis Induction Through Lipid Metabolism Modulation

Ferroptosis is a recently characterized form of cell death driven by iron-dependent lipid peroxidation, mechanistically distinct from apoptosis, necrosis, and autophagy [42,43,44]. In recent years, natural products have proved to induce ferroptosis in tumor cells through lipid peroxidation [45]. Cancer cells with high rates of lipid metabolism may be susceptible to ferroptosis induction, making this pathway an attractive therapeutic target [20,38].

Melanoma-related in vivo and in vitro experiments showed that the mitochondrial dysfunction and reactive oxygen species (ROS)-dependent ferroptosis were induced after salidroside intervention. Exposure of A-375 cells to salidroside elevated lipid peroxidation and triggered mitochondrial impairment, and then lipid ROS and malondialdehyde were generated, promoting ferroptosis in A-375 cells [20].

In triple-negative breast cancer, salidroside was shown to sensitize cancer cells to ferroptosis through two interconnected mechanisms [38]. By inhibiting SCD1-mediated lipogenesis, salidroside suppressed the PI3K/Akt/mTOR pathway, leading to downregulation of stearoyl-CoA desaturase 1 (SCD1). SCD1 catalyzes the rate-limiting step in monounsaturated fatty acid synthesis, and its inhibition results in increased lipid peroxidation and ROS-dependent ferroptosis susceptibility [38].

These findings establish a link between salidroside’s effects on lipid metabolism and its ability to induce ferroptosis in cancer cells.

## 4. Other Signaling Pathways Involved in Salidroside’s Metabolic Effects

### 4.1. SLC7A11/Cystine/GSH/GPX4 Pathway

The function of cystine/glutamate antiporter-solute carrier family 7 member 11 (SLC7A11) is to import cystine for glutathione biosynthesis and antioxidant defense, which is overexpressed in some cancers [20,46,47]. Recent studies have revealed that SLC7A11 overexpression promotes tumor growth partly through suppressing ferroptosis associated with SLC7A11-mediated metabolic reprogramming, leading to glucose and glutamine dependency in cancer cells [47], which indicates potential metabolic vulnerabilities for cancer target therapy.

In lung adenocarcinoma, salidroside targeted and downregulated SLC7A11, disrupting cystine uptake and glutathione (GSH) metabolism, leading to glutathione peroxidase 4 (GPX4) inactivation, and induced ferroptosis to inhibit cancer cell proliferation, while SLC7A11 overexpression reversed salidroside-mediated ferroptosis and anti-tumor activity. Concurrently, salidroside suppressed the AKT/mTORC1 signaling pathway to lower GPX4 expression, and mTOR activator counteracted salidroside’s efficiency [47].

### 4.2. MEK/ERK and HIF-1α Pathway

The MEK/ERK pathway and its downstream effector HIF-1α are central regulators of the Warburg effect, promoting glycolysis under both normoxic and hypoxic conditions [48]. HIF-1α is an important transcriptional regulator of glycolytic genes, including GLUT1, HK2, and LDHA [49]. In lung and pancreatic cancer, salidroside represses proliferation, migration, and invasion of cancer cells through inhibiting the MEK/ERK and HIF-1α pathways [50,51]. By suppressing the pathways, salidroside is speculated to reduce glycolysis in cancers, indicating another possible mechanism of metabolic regulation by salidroside.

## 5. Preclinical Evidence for Salidroside’s Anti-Tumor Effects

### 5.1. Preclinical Studies and Clinical Translation

Salidroside, a phenylethanol glycoside and principal bioactive component of *Rhodiola rosea*, has been substantiated to have multi-target effects against various malignancies. Studies have shown that salidroside presents broad anti-tumor activity against malignancies, including gastric, colon, liver, pancreatic, lung, breast, cervical, ovarian, and prostate cancers [22].

There is no research thus far on the pharmacokinetics of salidroside in humans. In vivo experiments showed that the half-life (T1/2) of salidroside was 0.70 ± 0.21 h after intravenous administration at a dose of 50 mg/kg and 1.32 ± 0.22 h after oral administration at a dose of 100 mg/kg in rats; however, the bioavailability of salidroside through oral administration decreased to 51.97% [52]. Pharmacokinetic studies suggest that salidroside has a short half-life and low oral bioavailability, mainly being metabolized in the liver and eliminated via renal excretion [52,53,54]. Almost no adverse reactions were reported in the animal studies. In vivo and in vitro studies showed that salidroside suppresses the growth and progression of cancers through inhibiting tumor cell proliferation, arresting the cell cycle, and decreasing cell migration and invasion. The metabolic anti-tumor effects of salidroside involved regulating oxidative stress, promoting lipid-peroxidation-mediated ferroptosis and mitochondrial dysfunction, inhibiting glycolysis and the lipid metabolism axis, and regulating key signaling pathways such as JAK2/STAT3, PI3K/Akt/mTOR, and MEK/ERK/ HIF-1α. Key findings from studies of salidroside regulating tumor energy metabolism are summarized in Table 1.

In terms of clinical translation, salidroside lacks sufficient clinical reports. Only a small-sample clinical study reported that the application of salidroside could attenuate epirubicin-induced cardiotoxicity in patients with breast cancer. In the study, salidroside was shown to have a cardiovascular protective effect on epirubicin-induced early left ventricular regional systolic dysfunction in breast cancer patients [55]. Nevertheless, the efficacy and safety of salidroside need careful evaluation to conduct clinical trials on cancers. Furthermore, appropriate strategies to optimize the therapeutic protocol for the application of salidroside in complex disease backgrounds still require meticulous design.

### 5.2. Challenges for Future Research and Clinical Translation

Despite encouraging preclinical studies, several challenges remain for future research and clinical translation of salidroside as a metabolic-targeting anti-tumor agent.

One challenge is the pharmacokinetic and bioavailability limitations. Salidroside has relatively low oral bioavailability due to its extensive first-pass metabolism and rapid clearance [53,54,56]. Strategies to improve bioavailability are under investigation, such as developing targeted delivery systems, which are regarded as effective strategies to enhance bioavailability [57]. A study showed that poly (lactic-co-glycolic acid) (PLGA)-based nanoparticles (NPs) modified with tumor-homing peptides (iVR1 peptides) and co-loaded with salidroside and apatinib (iVR1-NPs-Apa/Sal) realized highly efficient tumor-targeting drug delivery, regulating the tumor hypoxia microenvironment and enhancing the chemotherapeutic efficacy of drug-resistant cancer [58]. Another study showed that an engineered red blood cell membrane (RBCm)-coated salidroside/indocyanine green nanovesicle (ARISP) induces hypoxic-targeting phototherapy in triple-negative breast cancer. Salidroside in ARISP effectively ameliorates hypoxia-induced tumorigenesis by downregulating HIF-1α expression and significantly inhibits lung and liver metastasis in breast cancer models [59]. In the future, combining salidroside with functional materials may achieve stable drug release and enhanced bioavailability, providing a strategy for further exploration. Furthermore, forthcoming studies should be conducted on innovative synthesized drugs, elucidating their overall mechanisms and systematically assessing their therapeutic efficacy on cancers, which may provide some directions for promoting salidroside-based cancer treatment.

Another challenge is to maximize salidroside’s anti-tumor efficacy while minimizing its adverse effects. Although some studies demonstrated that salidroside-based delivery systems can enhance anti-tumor efficacy, research on the safety of such platforms remains limited [60]. To avoid possible adverse effects, safety should be given serious consideration. Although no serious adverse effects have been reported in current studies, their relatively short treatment durations, 14–28 days in these preclinical models, limit the availability of long-term safe therapy. Therefore, achieving a safe therapeutic treatment interval with high efficacy and low toxicity remains a significant challenge.

Salidorside dosage is another issue to consider, as there is a range of effective dosages reported in animal studies. Table 1 summarizes the doses administered both in vitro and in vivo, which exhibit variability. This heterogeneity is probably attributable, at least in part, to differences in drug sensitivity among tumor types. Given that dosage is a critical factor for clinical translation, studies on appropriate dosage application should be investigated before clinical trials [61].

To date, the most pressing challenge is that no randomized clinical trials have been conducted to evaluate salidroside in patients with cancer. Therefore, no clinical evidence confirms salidroside’s metabolic-targeted anti-tumor effects in cancer patients, and human-equivalent dose conversions have not been provided yet, perhaps because the pharmacokinetic and bioavailability limitations in the human body are not clear. There may be a feasible way to estimate the dosage for humans based on the established dosage from animals. However, different cancer types have different salidroside doses administrated in vivo and in vitro as shown in Table 1. The salidroside dosage may be dependent on the drug sensitivity of the cancer type. To avoid possible potential hazards and drug resistance, serious consideration should be given to safe and appropriate dosages in clinical trials, especially for cancer patients with complex comorbidities. These trials are required to evaluate the safety, tolerability, pharmacokinetic profiles, and potential drug resistance of salidroside in human subjects. Since the heterogeneity of metabolism progresses, cancer cells may develop compensatory metabolic pathways to overcome salidroside’s effects. Alternatively, tumor metabolic phenotypes vary widely across tumor types and even within individual tumors. Therefore, it is necessary to conduct integrated multi-omics analysis. Comprehensive metabolomic, lipidomic, and proteomic analyses of salidroside-treated tumors will provide a complete framework of metabolism.

The selected biomarkers may be used to detect patients with cancer at first, and based on current mechanisms, predictive biomarkers may be identified for cancers. Research indicates that high OGDHL expression is associated with colon and liver cancer progression [62,63]. Similarly, studies have found that PDHB is highly expressed in various cancer cells such as gastric, breast, kidney, and liver cancer [8,64,65,66]. In ovarian cancer, OGDHL and PDHB are significantly upregulated, indicating that their high expression promotes the growth of cancer cells through increasing glycolysis and ATP production [67]. In terms of ACOT11, studies indicate that high ACOT11 expression promotes cell proliferation, migration, and invasion in gastric and lung cancer [9,68]. Although some studies have shown that miR-1343-3p is downregulated in gastric, colon, and lung cancer, indicating it may inhibit the proliferation and migration of cancer cells [8,69,70], only a few studies have shown that miR-1343-3p may promote docetaxel resistance in breast cancer and inhibit the autophagy in multiple myeloma [71,72]. The different effects of miR-1343-3p on cancers suggest that this miRNA may be target- and tissue-context-dependent. Therefore, miR-1343-3p should be evaluated before clinical application.

Studies indicate that salidroside has multiple effects such as antioxidant, anti-fatigue, and anti-aging activity. Therefore, it may be suitable for adjuvant therapy in clinical trials. For example, salidroside ameliorated cisplatin-induced nephrotoxicity in ovarian cancer and conferred protective activity against doxorubicin-induced cardiomyopathy in an in vivo mouse experiment [73,74]. In cancer cachexia mouse models, salidroside protected the body from weight loss and prolonged survival [75].

Combination strategies targeting multiple pathways are likely to be beneficial to cancer treatment. In contrast to conventional single-target drugs, salidroside exerts its effects towards multiple targets, thereby reducing the possibility of tumor drug resistance. One study showed that salidroside overcame cisplatin resistance in ovarian cancer by inhibiting autophagy, and activating the PI3K/AKT/mTOR pathway, which is related to lipid peroxidation and ferroptosis [73]. Additionally, these strategies may be expanded to address more cancer types. Given the main function of metabolic organs, salidroside may have efficacy in metabolically driven cancers such as hepatocellular carcinoma, pancreatic adenocarcinoma, and renal cancer.

Salidroside may also increase the efficacy of immunotherapy. In lung cancer, salidroside suppressed cancer cell proliferation, migration, invasion, and immune escape in vitro via regulating the circ_0009624/PD-L1 pathway [76]. When combined with chemotherapy or radiotherapy, salidroside may attenuate toxicity and adverse effects while enhancing the sensitivity of cancer cells to oxaliplatin, doxorubicin and paclitaxel [77,78,79].

To summarize, combination therapy may be the optimum strategy for future clinical application. Overall, salidroside demonstrates therapeutic efficacy in multiple cancers through established mechanisms, highlighting its potential advantage in cancer therapy.

## 6. Conclusions

Recently, substantial progress has been made in understanding how salidroside regulates tumor energy metabolism to suppress cancer cell proliferation. The findings of miR-1343-3p targeting glucose metabolism through the OGDHL/PDHB axis and lipid metabolism through the ACOT11 axis provide a mechanism of how this natural compound exerts its anti-tumor effects in gastric cancer. Furthermore, salidroside inhibits the proliferation of some cancers through the STAT3/c-Myc pathway, reducing glucose consumption, lactate production and glycolytic enzyme expression. Salidroside also regulates lipid metabolism to sensitize cancer cells to ferroptosis via lipid peroxidation. Other signaling pathways, MEK/ERK and PI3K/Akt/mTOR, mediate salidroside’s anti-tumor effects, providing additional viewpoints for cancer metabolic regulation. However, clinical translation still faces many challenges including limited bioavailability, lack of clinical trial data, and potential for safety and adverse effects. Further research should focus on pharmacokinetic exploration, drug delivery system development, and optimization of combination therapy strategies to realize the clinical application of salidroside. With our deepening understanding of tumor metabolism going further in depth, salidroside is likely to be an optimal tool for cancer therapy.

## Figures and Tables

**Table 1:** Key preclinical studies of salidroside regulating tumor energy metabolism.

Cancer Type	Model	SAL Dosage, Usage, and Administration Route	Metabolic Endpoint	Mechanisms	Outcomes of SAL Administration	Reference	Note
Gastric cancer	In vivo/BALB/c nude mice with MGC-803 cells and in vitro/MGC-803 cells from humans	SAL 100 mg/kg, i.p., every day for 28 d (for in vivo) and 4, 6, and 8 mM for 48 h (for in vitro)	Pyruvic acid↑ Acetyl-CoA↓ ATP↓	miR-1343-3p↑ OGDHL protein↓ PDHB protein↓	Cell proliferation↓ Cell colony formation↓ Cell migration↓ Tumor volume and weight↓	[8]	↑ Increase ↓ Decrease
Gastric cancer	In vivo/BALB/c nude mice with MGC-803 cells and in vitro/MGC-803 cells from humans	SAL 100 mg/kg, i.p., every day for 28 d (for in vivo) and 4, 6, and 8 mM for 48 h (for in vitro)	Fatty acyl-CoA↑ Acetyl-CoA↓ FFA↓ ATP↓	miR-1343-3p↑ ACOT11 protein↓	Cell proliferation↓ Cell colony formation↓ Cell migration↓ Tumor volume and weight↓	[9]	↑ Increase ↓ Decrease
Ovarian cancer	In vivo/BALB/c nude mice with SKOV3 cells and in vitro/CAOV3 and SKOV3 cells from humans	SAL 80 mg/kg, i.p., once every two days for 20 d (for in vivo) and 200, 400, and 800 μM for 24 h (for in vitro)	GLUT1, HK2, and LDHA protein↓ Glycolysis↓	STAT3/c-Myc pathway↓ Phosphorylated STAT3 and c-Myc proteins↓	Cell proliferation↓ Cell colony formation↓ Cell migration and invasion↓ Tumor volume and weight↓	[34]	↑ Increase ↓ Decrease
Triple-negative breast cancer	In vivo/BALB/c nude mice with MDA-MB-231 cells and in vitro/MDA-MB-231 cells from humans	SAL 100 mg/kg, axilla injection, once daily for 14 d (for in vivo) and 50 μM for 24 h (for in vitro)	Lipid peroxidation↑ Lipogenesis of monounsaturated fatty acid↓	PI3K/Akt/mTOR pathway↓ Fe^2+^↑ ROS-dependent ferroptosis↑	Cell proliferation↓ Tumor volume and weight↓	[38]	↑ Increase ↓ Decrease
Melanoma	In vivo/C57BL/6J mice with A-375 cells and in vitro/A-375 cells from humans	SAL 100 mg/kg, p.o., per day for 3 weeks (for in vivo) and 100, 200, and 400 mM for 24 h (for in vitro)	Glycolysis↓ Lipid peroxidation↑	Mitochondrial respiratory chain complexes↓ SLC7A11 and GPX4 protein↓ Mitochondrial dysfunction↑ ROS-dependent ferroptosis↑	Cell proliferation↓ Cell colony formation↓ Cell migration and invasion↓ Tumor volume and weight↓	[20]	↑ Increase ↓ Decrease
Lung cancer	In vivo/BALB/c nude mice with A549 cells and in vitro/A549 and H1975 cells from humans	SAL 100 mg/kg, i.p., once daily for 16 d (for in vivo) and 10, 20, 40, 80, and 160 μM for 24 h (for in vitro)	Lipid peroxidation↑	SLC7A11/ cystine/GSH/GPX4 pathway↓ AKT/mTORC1/GPX4 pathway↓ Fe^2+^↑ ROS-dependent ferroptosis↑	Cell proliferation↓ Cell colony formation↓ Cell migration and invasion↓ Tumor volume and weight↓	[47]	↑ Increase ↓ Decrease

## Data Availability

The data that support the findings of this study are available from the corresponding author upon reasonable request.
